# Trends in Pleural Mesothelioma Incidence and Survival in Metropolitan and Nonmetropolitan Areas in the United States

**DOI:** 10.1002/cam4.71474

**Published:** 2025-12-15

**Authors:** Alexander J. Didier, Charlotte Lennox, Mingjia Li, Jinesh Gheeya, Asrar Alahmadi, Jacob Kaufman, Regan Memmott, Kai He, Peter Shields, Christian Rolfo, David P. Carbone, Carolyn Presley, Dwight Owen, Logan Roof

**Affiliations:** ^1^ Department of Medicine The University of Toledo College of Medicine and Life Sciences Toledo Ohio USA; ^2^ Division of Medical Oncology, Department of Internal Medicine The Ohio State University Columbus Ohio USA

## Abstract

**Introduction:**

Pleural mesothelioma (PM) is a rare, aggressive cancer with significant variation in incidence based on geographic factors. Previous studies have highlighted cancer survival disparities between metropolitan and nonmetropolitan populations for other cancers; this data is largely unreported for PM. We aimed to compare incidence trends and cancer‐specific survival (CSS) between metropolitan and nonmetropolitan areas in the United States using the Surveillance, Epidemiology, and End Results (SEER) database.

**Methods:**

We analyzed SEER 18 registries for patients aged ≥ 20 diagnosed with PM between 2004 and 2021. Incidence rates, CSS, and demographic and clinical characteristics were compared between metropolitan and nonmetropolitan areas. Incidence rate ratios (IRRs) were calculated using the Tiwari method. Joinpoint regression was used to assess temporal trends, while Kaplan–Meier and Cox proportional hazard models analyzed survival outcomes.

**Results:**

A total of 8519 PM cases were identified, with 89.3% in metropolitan areas. Nonmetropolitan patients were more likely to be non‐Hispanic Black and had lower chemotherapy (*p* = 0.031) and surgery (*p* < 0.001) rates. The incidence rate in metropolitan areas declined from 1.4 in 2004 to 0.8 in 2021, while nonmetropolitan areas saw a stable incidence until 2017, followed by a decline to 0.5 in 2021. Metropolitan areas had significantly higher CSS, with 50.3% 1‐year CSS by 2020, compared to 27.7% in nonmetropolitan areas. Multivariate analysis indicated a higher hazard of death in nonmetropolitan areas (HR = 1.18, *p* < 0.001).

**Conclusion:**

Significant disparities in PM outcomes between metropolitan and nonmetropolitan areas were revealed. Although both regions experienced a decline in incidence over time, survival outcomes remained worse in nonmetropolitan areas. Patients in nonmetropolitan areas were also less likely to receive chemotherapy and surgery, further contributing to the survival gap. These findings highlight the need for targeted interventions to improve treatment access and enhance survival for nonmetropolitan patients with PM.

## 1 Introduction

Pleural mesothelioma (PM) is the most common subtype of malignant mesothelioma, accounting for 90% of all cases [1]. The most common PM risk factor is asbestos exposure, most often through an occupational hazard or the ambient environment [2, 3]. PM survival rates have generally been poor, with only 5%–10% survival at 5 years after diagnosis [1]. Historically, PM treatment options have been limited, with most patients receiving a combination of pemetrexed and platinum‐based chemotherapies. Recent clinical trials exploring the use of immunotherapy have shown promising results, expanding the frontline systemic therapy options available to patients [1, 4].

There is increasing evidence of disparities in incidence and survival for patients with cancer in nonmetropolitan regions compared with their metropolitan counterparts, which may be related to intersecting factors including older age; lower educational status; and higher prevalence of obesity, smoking, and other behavioral risk factors [5]. Additionally, patients residing in rural areas experience lower access to healthcare and academic institutions, which are important centers of care for patients with PM [5]. Rural populations have experienced slower decreases in age‐adjusted mortality, especially for leading causes of cancer mortality such as lung and breast cancer [6]. This led to an initiative launched in 2018 by the American Society of Clinical Oncology aimed at understanding the factors associated with rural cancer disparities [5]. Studies have suggested that metropolitan regions may have a higher PM incidence than nonmetropolitan regions due to increased historical usage of asbestos and occupational exposure to asbestos in these areas [7, 8]. Given declines in occupational asbestos exposure throughout the past several decades and the United States (US) Environmental Protection Agency (EPA) ban on asbestos in 2023, the gap between metropolitan and nonmetropolitan PM incidence may be closing as PM incidence is expected to decrease, although this data has not been thoroughly explored in the literature [9, 10]. Since 2013, PM incidence has decreased at a rate of −5.2% annually and is expected to continue to decrease [10]. Since there is a long latency period of 30–40 years between asbestos exposure and PM development, it may take time to witness these changes. For PM, data comparing the incidence and survival between regions of differing geographic density have not been reported. To address this gap, our study explicitly compares cancer incidence and survival trends between metropolitan and nonmetropolitan areas, where limited comparative data currently exist.

This study aims to identify current PM incidence and survival trends in metropolitan and nonmetropolitan areas of the US. We further compare demographic and clinical characteristics between these groups to explore relationships between these characteristics and survival in patients with PM. Understanding these trends can reveal patterns in healthcare access, treatment response, and disease progression. This allows healthcare providers and policymakers to design targeted interventions, such as improving early detection programs in underserved areas, allocating resources to high‐risk populations, and customizing treatment plans based on specific population needs. This tailoring helps ensure that care is both equitable and effective across diverse geographic and demographic groups.

## 2 Materials and Methods

### 2.1 Dataset

We queried the Surveillance, Epidemiology, and End Results (SEER) 18 (SEER 18) registries for all cases of pleural mesothelioma diagnosed between 2004 and 2021 [11]. SEER 18 encompasses approximately 34% of the US population and is considered the gold standard for population‐based studies assessing cancer incidence and survival in the US [11]. Pleural mesothelioma cases were identified using International Classification of Diseases, Tenth Revision (ICD‐10) codes corresponding with primary site of the lung (C34.0–C34.9) or pleura (C48.9), as well as ICD‐10 histology codes encompassing malignant mesothelioma (9050/3, 9051/3, 9052/3, 9055/3). We included patients ≥ 20 years of age at diagnosis with microscopically confirmed PM. Patients < 20 years of age, those without survival information, and those without information related to metropolitan status were excluded. Our dataset (SEER 18) includes cancer cases from 18 registries across the US, including Alaska, Greater Georgia, Connecticut, Detroit (metropolitan), Hawaii, Iowa, New Mexico, Rural Georgia, California excluding San Francisco‐Oakland/San Jose‐Monterey/Los Angeles, San Francisco‐Oakland, San Jose‐Monterey, Seattle, Utah, Kentucky, Los Angeles, Louisiana, New Jersey, and Atlanta (metropolitan). Individual patient‐level and population‐level data from the SEER database were extracted using SEER*Stat software (version 8.4.3; Surveillance Research Program of the National Cancer Institute; RRID:SCR_025808) [11].

### 2.2 Variables

We included several demographic and clinical variables in our analysis. Patient age was grouped into four categories: 20–39, 40–59, 60–79, and ≥ 80 years old. Race/ethnicity was defined as non‐Hispanic White, non‐Hispanic Black (NHB), non‐Hispanic Asian or Pacific Islander, non‐Hispanic American Indian/Alaskan Native, and Hispanic, per the SEER database. Marital status was categorized as single, married, divorced, widowed, or unknown. Sex was defined as male or female. Receipt of chemotherapy and radiotherapy were defined as “yes” or “no/unknown,” per the SEER database. Receipt of surgery was categorized into a binary “yes” or “no/unknown.” Histology was categorized as mesothelioma, fibrous, epithelioid, and biphasic, per the SEER database. Stage was classified using the SEER summary stage variable, which includes localized, regional, distant, and unknown stage.

Metropolitan counties were defined as those in metropolitan statistical areas (MSAs, or a geographic region including a city or urban area and its surrounding communities) with populations ≥ 1 million; large fringe metro counties in MSAs with populations of ≥ 1 million that do not qualify as large central or medium metro counties in MSAs of 250,000–999,999 population; and small metro counties in MSAs with populations < 250,000. Nonmetropolitan counties were defined as micropolitan counties in micropolitan statistical areas and noncore counties not in micropolitan statistical areas. Micropolitan statistical areas are associated with at least 1 urban cluster of a population of 10,000–49,999; micropolitan statistical areas also include adjacent counties having a high degree of social and economic integration with the core as measured through commuting ties [12].

### 2.3 Statistical Analysis

We first compared proportions of demographic and clinical characteristics of PM between metropolitan and nonmetropolitan regions using Chi‐squared analysis. Next, we compared incidence rates and trends between these groups. Incidence rates were calculated as the number of new cases diagnosed each year per 100,000 individuals [13]. Incidence rate ratios (IRRs) were calculated using the Tiwari method to compare differences between metropolitan and nonmetropolitan areas [14]. The IRR represents the ratio of the number of new PM cases diagnosed in nonmetropolitan areas divided by the number of those diagnosed in metropolitan areas. Joinpoint regression analysis was performed to identify statistically significant temporal changes using Joinpoint software (National Cancer Institute, Bethesda, MD; RRID: SCR_018129) [15]. This method separates the data into joinpoints, which represent years where the trend in mortality shifted. Joinpoint regression utilizes a Monte Carlo permutation method to determine whether changes in trendlines are statistically significant, meaning that there was an increase or decrease in the annual percentage change of mortality that was different from zero [15]. Finally, we examined cancer‐specific survival (CSS) differences between metropolitan and nonmetropolitan areas. CSS is calculated as the survival percentage at a prespecified time interval in the absence of death due to cancer and is a commonly utilized method of estimating survival. We elected to investigate 1‐ and 5‐year CSS in this study to comprehensively compare differences in survival in this patient population. Propensity score matching using 1:1 matching with nearest neighbors method was utilized to control for demographic and clinical characteristics that may influence survival. Kaplan–Meier survival analysis and the log‐rank test were performed to compare CSS between metropolitan and nonmetropolitan groups before and after propensity score matching. Additionally, Cox proportional hazards models were developed to obtain hazard ratios (HRs) for death due to PM. Univariate analysis was performed, with variables with *p* < 0.10 selected for inclusion in the multivariate analysis. All statistical tests were 2‐tailed, and *p* ≤ 0.05 was considered statistically significant. Data analysis was performed in September 2024 using SPSS software (Chicago, IL; RRID: SCR_002865).

## 3 Results

### 3.1 Characteristics of the Study Population

Between 2004 and 2021, 8519 cases of PM were diagnosed in the US and met inclusion criteria for the study (Table 1). Most patients resided in metropolitan areas (*n* = 5788, 89.3%). Patients in rural nonmetropolitan areas were more likely to be NHW (88.9% vs. 77.2%, *p* < 0.001) and less likely to receive chemotherapy (48.6% vs. 44.8%, *p* = 0.031) and surgery (24.4% vs. 20.2%, *p* < 0.001). There were no significant differences in age at diagnosis, histologic subtype, stage at diagnosis, or receipt of radiation between groups.

**TABLE 1:** Sample characteristics.

Characteristic	Metropolitan (*n* = 7607, 89.3%)	Nonmetropolitan (*n* = 912, 10.7%)	*p*
*Demographic*			
Sex			0.459
Male	5788 (76.1%)	704 (77.2%)	
Female	1819 (23.9%)	208 (22.8%)	
Race/ethnicity			< 0.001
NHW	5869 (77.2%)	811 (88.9%)	
NHB	379 (5.0%)	36 (3.9%)	
NH Asian	317 (4.2%)	6 (0.7%)	
NH AI/AN	31 (0.4%)	10 (1.1%)	
Hispanic	994 (13.1%)	49 (5.4%)	
Unknown	17 (0.2%)	0 (0.0%)	
Age			0.072
20–39	61 (0.8%)	5 (0.5%)	
40–59	889 (11.7%)	109 (12.0%)	
60–79	4480 (58.9%)	572 (62.7%)	
80+	2177 (28.6%)	226 (24.8%)	
Marital status			0.033
Married	4851 (63.8%)	604 (66.2%)	
Divorced	616 (8.1%)	86 (9.4%)	
Single	762 (10.0%)	64 (7.0%)	
Widowed	1090 (14.3%)	123 (13.5%)	
Unknown	288 (3.8%)	35 (3.8%)	
*Clinical*			
Histology			0.657
Mesothelioma	3232 (42.5%)	373 (40.9%)	
Fibrous mesothelioma	880 (11.6%)	111 (12.2%)	
Epithelioid mesothelioma	2875 (37.8%)	359 (39.4%)	
Biphasic mesothelioma	620 (8.2%)	69 (7.6%)	
Stage			0.606
Localized	791 (10.4%)	92 (10.1%)	
Regional	1405 (18.5%)	154 (16.9%)	
Distant	5026 (66.1%)	622 (68.2%)	
Unknown	385 (5.1%)	44 (4.8%)	
Received radiation			0.223
Yes	879 (11.6%)	93 (10.2%)	
No/unknown	6728 (88.4%)	819 (89.8%)	
Received chemotherapy			0.031
Yes	3699 (48.6%)	409 (44.8%)	
No/unknown	3908 (51.4%)	503 (55.2%)	
Received surgery			0.005
Yes	1857 (24.4%)	184 (20.2%)	
No/unknown	5750 (75.6%)	728 (79.8%)	

Abbreviations: AI/AN, American Indian/Alaskan Native; NH, non‐Hispanic; NHB, non‐Hispanic Black; NHW, non‐Hispanic White.

### 3.2 Trends in Incidence

**FIGURE 1:**
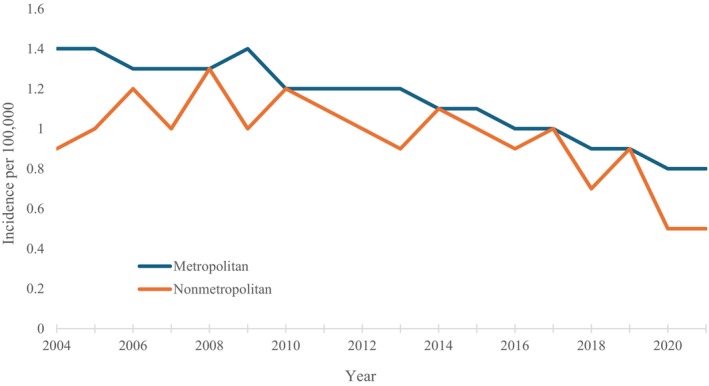
Pleural mesothelioma incidence trends in metro and nonmetro areas: 2004–2021. Trends in pleural mesothelioma incidence in metropolitan and nonmetropolitan areas between 2004 and 2021.

Between 2004 and 2021, metropolitan areas experienced an average age‐adjusted incidence rate of 1.1, higher than nonmetropolitan areas with an incidence of 0.9 (IRR: 0.85, *p* < 0.001). For both groups, incidence rates declined over the study period (Figure 1). Metropolitan areas experienced a decline in incidence from 1.4 in 2004 to 1.2 in 2013 at −1.8% annually (95% confidence interval [CI] −2.8%, 0.8%). Beginning in 2014, metropolitan areas saw a steeper decline to 0.8 in 2021, corresponding to −4.9% each year (95% CI −8.1%, −3.9%). For nonmetropolitan areas, incidence remained stable at 0.9 from 2004 to 2017, after which it declined to 0.5 in 2021 at a rate of −16.2% annually (95% CI −40.4%, −6.0%).

### 3.3 Survival Analysis

**FIGURE 2:**
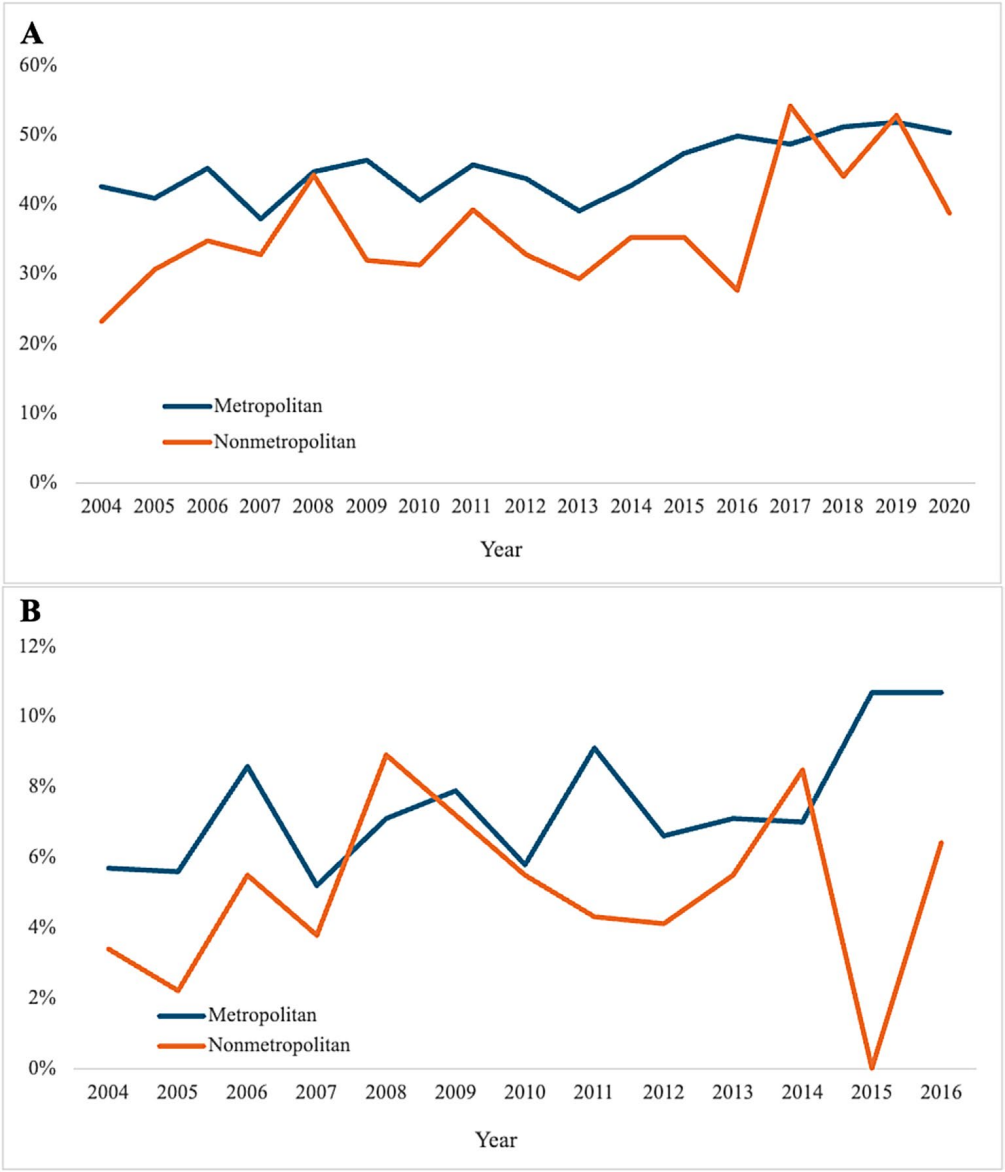
Cancer‐specific survival trends in patients with PM in metropolitan and nonmetropolitan areas. Trends in (A) 1‐year and (B) 5‐year cancer‐specific survival in patients with pleural mesothelioma in metropolitan and nonmetropolitan areas.

**FIGURE 3:**
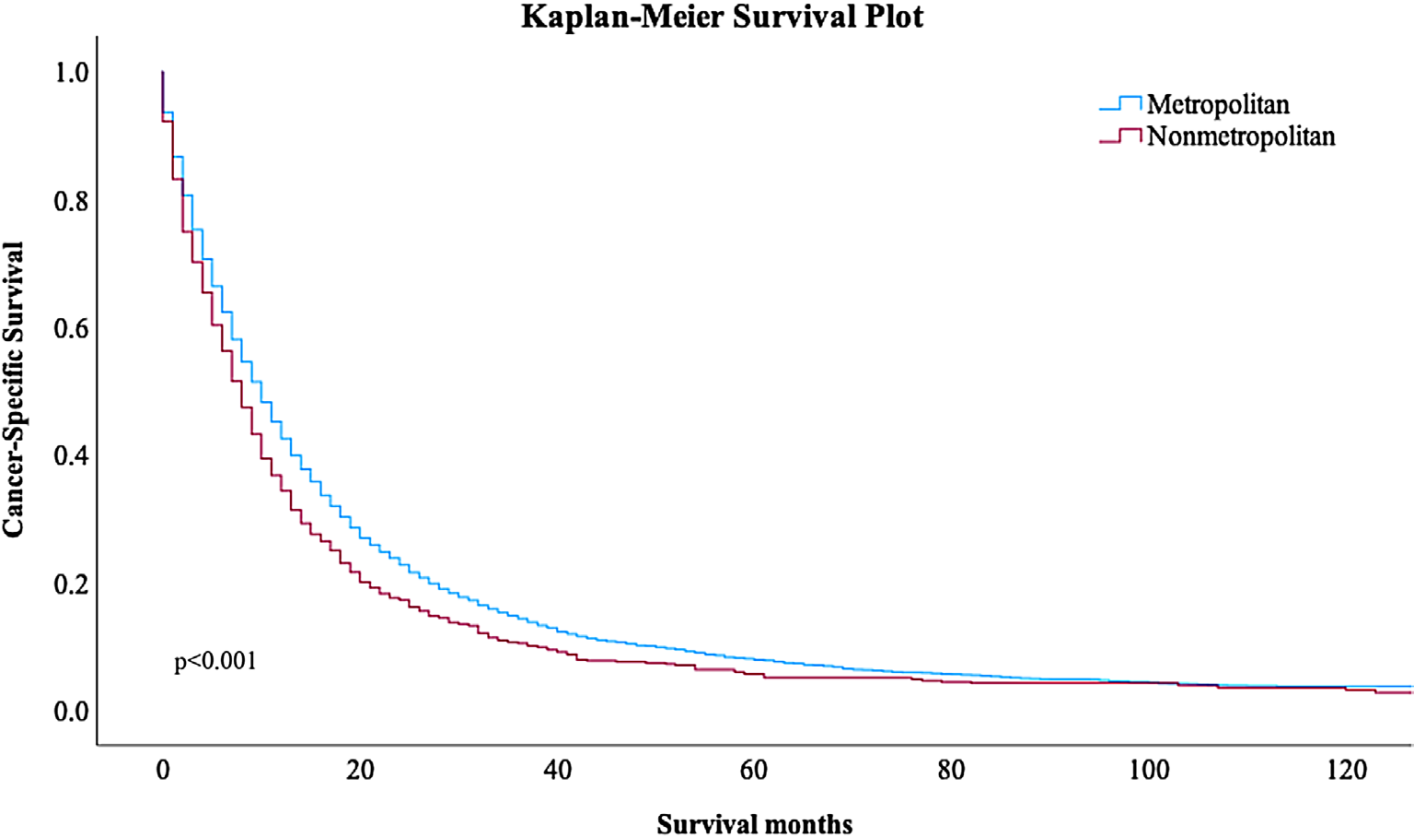
Kaplan–Meier survival analysis comparing metropolitan and nonmetropolitan areas. Kaplan–Meier survival analysis and log rank test comparing metropolitan and nonmetropolitan areas, log rank test *p* < 0.001.

Metropolitan areas experienced significantly higher CSS than nonmetropolitan areas across the study period, and both 1‐ and 5‐year CSS improved between 2004 and 2021 (Figure 2). In 2004, metropolitan areas experienced 42.6% 1‐year CSS and 5.7% 5‐year CSS, almost twice as high as that of nonmetropolitan areas with a 1‐year CSS of 23.1% and 5‐year CSS of 3.4%. By 2020, 1‐year CSS had improved in both groups and the gap had narrowed, with metropolitan areas experiencing a 50.3% 1‐year CSS compared with 27.7% in nonmetropolitan areas. By 2016, the last year we were able to examine 5‐year CSS, similar findings were noted. Five‐year CSS was 10.7% for metropolitan areas and 6.4% for nonmetropolitan areas. After propensity score matching, there were two equal cohorts of 912 patients (Table 2). One‐year CSS was 44.4% in the metropolitan cohort compared with 34.3% in the nonmetropolitan cohort, while 5‐year CSS was 8.8% in the metropolitan cohort and 5.6% in the nonmetropolitan cohort. Results of the Kaplan–Meier survival analysis and log‐rank test can be found in Figure 3. Multivariate Cox proportional hazard analysis showed greater hazard of death for individuals living in nonmetropolitan areas (HR = 1.18, 95% CI 1.09–1.27, *p* < 0.001) (Table 3). Other variables associated with increased hazard of death included male sex (HR = 1.23, 95% CI 1.16–1.31, *p* < 0.001), fibrous (HR = 1.68, 95% CI 1.55–1.81, *p* < 0.001) and biphasic (HR = 1.28, 95% CI 1.17–1.40, *p* < 0.001) histology, advanced stage at diagnosis (HR = 1.37, 95% CI 1.27–1.49, *p* < 0.001), and lack of receipt of chemotherapy (HR = 1.38, 95% CI 1.31–1.50, *p* < 0.001) or surgery (HR = 1.41, 95% CI 1.33–1.50, *p* < 0.001).

**TABLE 2:** Propensity score matching.

Characteristic	Metropolitan (*n* = 912, 50.0%)	Nonmetropolitan (*n* = 912, 50.0%)	*p*
*Demographic*			
Sex			0.233
Male	725 (79.5%)	704 (77.2%)	
Female	187 (20.5%)	208 (22.8%)	
Race/ethnicity			0.054
NHW	838 (91.9%)	811 (88.9%)	
NHB	35 (3.8%)	36 (3.9%)	
NH Asian	4 (0.4%)	6 (0.7%)	
NH AI/AN	2 (0.2%)	10 (1.1%)	
Hispanic	33 (3.6%)	49 (5.4%)	
Unknown	0 (0%)	0 (0%)	
Age			0.358
20–39	1 (0.1%)	5 (0.5%)	
40–59	100 (11.0%)	109 (12.0%)	
60–79	586 (64.3%)	572 (62.7%)	
80+	225 (24.7%)	226 (24.8%)	
Marital status			0.259
Married	648 (71.1%)	604 (66.2%)	
Divorced	72 (7.9%)	86 (9.4%)	
Single	59 (6.5%)	64 (7.0%)	
Widowed	106 (11.6%)	123 (13.5%)	
Unknown	27 (3.0%)	35 (3.8%)	
*Clinical*			
Histology			0.692
Mesothelioma	364 (39.9%)	373 (40.9%)	
Fibrous mesothelioma	99 (10.9%)	111 (12.2%)	
Epithelioid mesothelioma	381 (41.8%)	359 (39.4%)	
Biphasic mesothelioma	68 (7.5%)	69 (7.6%)	
Stage			0.711
Localized	86 (9.4%)	92 (10.1%)	
Regional	158 (17.3%)	154 (16.9%)	
Distant	633 (69.4%)	622 (68.2%)	
Unknown	35 (3.8%)	44 (4.8%)	
Received radiation			0.339
Yes	81 (8.9%)	93 (10.2%)	
No/unknown	831 (91.1%)	819 (89.8%)	
Received chemotherapy			0.541
Yes	422 (46.3%)	409 (44.8%)	
No/unknown	490 (53.7%)	503 (55.2%)	
Received surgery			< 0.001
Yes	249 (27.3%)	184 (20.2%)	
No/unknown	663 (72.7%)	728 (79.8%)	

**TABLE 3:** Univariate and multivariate hazard ratios.

Characteristic	Univariate HR	95% CI	*p*	Multivariate HR	95% CI	*p*
*Demographic*						
Geographic area						
Metropolitan	Ref			Ref		
Nonmetropolitan	1.19	1.11–1.28	< 0.001	1.18	1.09–1.27	< 0.001
Sex						
Female	Ref			Ref		
Male	1.25	1.19–1.33	< 0.001	1.23	1.16–1.31	< 0.001
Race/ethnicity						
NHW	Ref			Ref		
NHB	0.98	0.87–1.09	0.647	1.03	0.92–1.15	0.604
NH Asian	1.05	0.93–1.19	0.437	1.13	1.00–1.28	0.052
NH AI/AN	0.80	0.60–1.12	0.190	0.8	0.58–1.11	0.187
Hispanic	0.96	0.89–1.03	0.218	1.03	0.96–1.11	0.373
Unknown	0.35	0.16–0.78	0.010	0.39	0.17–0.86	0.02
Age						
20–39	Ref			Ref		
40–59	1.38	1.03–1.84	0.031	1.34	1.00–1.80	0.048
60–79	1.82	1.37–2.42	< 0.001	1.67	1.25–2.23	< 0.001
80+	2.75	2.06–3.66	< 0.001	2.15	1.61–2.87	< 0.001
Marital status						
Married	Ref			Ref		
Divorced	0.99	0.91–1.08	0.842	1.06	0.97–1.15	0.217
Single	1.06	0.98–1.15	0.141	1.14	1.05–1.24	0.002
Widowed	1.22	1.14–1.31	< 0.001	1.08	1.01–1.17	0.034
Unknown	0.98	0.87–1.11	0.786	0.96	0.85–1.09	0.545
*Clinical*						
Histology						
Mesothelioma	Ref			Ref		
Fibrous mesothelioma	1.58	1.47–1.71	< 0.001	1.68	1.55–1.81	< 0.001
Epithelioid mesothelioma	0.69	0.66–0.73	< 0.001	0.8	0.76–0.84	< 0.001
Biphasic mesothelioma	1.05	0.96–1.14	0.321	1.28	1.17–1.40	< 0.001
Stage						
Localized	Ref			Ref		
Regional	0.99	0.91–1.10	0.958	1.11	1.01–1.23	0.025
Distant	1.28	1.19–1.39	< 0.001	1.37	1.27–1.49	< 0.001
Unknown	1.16	1.02–1.32	0.025	1.07	0.94–1.22	0.307
Received radiation						
Yes	Ref			Ref		
No/unknown	1.23	1.14–1.32	< 0.001	1.01	0.94–1.09	0.786
Received chemotherapy						
Yes	Ref			Ref		
No/unknown	1.54	1.47–1.61	< 0.001	1.38	1.31–1.50	< 0.001
Received surgery						
Yes	Ref			Ref		
No/unknown	1.65	1.56–1.74	< 0.001	1.41	1.33–1.50	< 0.001

Abbreviations: AI/AN, American Indian/Alaskan Native; NH, non‐Hispanic; NHB, non‐Hispanic Black; NHW, non‐Hispanic White.

## 4 Discussion

Several important findings are reported in this study. First, patients with PM who reside in nonmetropolitan areas were less likely to receive chemotherapy and surgery despite similar stage at diagnosis compared with those residing in metropolitan areas. Second, metropolitan areas experienced a higher incidence of PM compared with nonmetropolitan areas throughout the study period, with incidence decreasing in both groups by 2021. Third, metropolitan areas experienced higher 1‐ and 5‐ year CSS across the study period, almost twice as high as that of nonmetropolitan areas. Fourth, multiple clinical and demographic variables were found to be associated with higher hazard of death, including nonmetropolitan residence, male sex, fibrous and biphasic histology, and advanced stage at diagnosis.

PM accounts for nearly 90% of malignant mesothelioma cases and has historically poor outcomes due to its frequent diagnosis at an advanced stage and aggressive development of disease [1, 16]. Despite the challenges that advanced stage diagnoses bring, the treatment landscape continues to change and adapt. The most significant risk factor for PM development is asbestos exposure, with 80% of patients having a known history [17]. This well‐established association has contributed to the regulations in asbestos use in the US since the 1980s by the Occupational Safety and Health Administration (OSHA) and EPA [18, 19]. After numerous modifications of its initial restrictions, the EPA finalized its ruling in March 2024 that henceforth prohibited the continued use of chrysotile asbestos in the US [19]. Despite this recent ban, asbestos will have to be slowly phased out of buildings and industry; thus, these changes will not take effect immediately. Further, asbestos is still routinely used in many parts of the world, leading to a persistent increase in death counts due to mesothelioma in these regions [20, 21]. Additionally, due to the long latency period—roughly 30–40 years—between asbestos exposure and PM development, cases are not expected to decrease for quite some time, emphasizing the importance of ongoing research to evolve the current treatment landscape [22].

Historically, PM treatment options have been limited. However, recent treatment advances have shown promise, with multimodal care involving evaluations for surgical resection, radiotherapy, and systemic therapy at high‐volume academic centers of excellence being the preferred treatment [17, 23] The CHECKMATE 743 trial demonstrated a significant improvement in overall survival with nivolumab plus ipilimumab when compared to standard‐of‐care platinum‐based chemotherapy, especially for non‐epithelioid subtypes, leading to the FDA approval of immunotherapy, revolutionizing the PM treatment landscape [24] This trial also demonstrated the demand for histology‐specific systemic treatment plans given the difference in treatment response among histological subtypes [4] Additionally, per the KEYNOTE‐483 study, the FDA recently approved pembrolizumab in addition to chemotherapy for the first‐line treatment of unresectable advanced or metastatic PM [25] Given these developments in the use of immunotherapy for PM treatment, further research is needed to identify additional treatment options for immune‐refractory populations [4] Following these pivotal advances, additional clinical trials of chemoimmunotherapy, new forms of immunotherapy, targeted therapies, and cellular therapies are ongoing and promising.

The results of our study demonstrate lower CSS in nonmetropolitan areas compared with metropolitan areas. Despite recent advancements in treatment and restrictions to asbestos exposure, certain populations remain at a higher risk of death due to pre‐existing healthcare disparities, including populations in rural communities as compared with urban areas [5]. Some of these disparities include higher rates of obesity, older age, and increased behavioral risk factors including smoking and alcohol use. In addition, rural populations experience an increased distance to academic centers of care that treat a high volume of mesothelioma cases, which are historically more likely to be in metropolitan areas [5]. In our study, 1‐ and 5‐year CSS for patients with PM who resided in nonmetropolitan regions were almost half that of their metropolitan counterparts, a disparity that remained present at the end of the study period. Previous studies have demonstrated strong associations between rural residence and poor cancer survival, with one meta‐analysis demonstrating that those living in rural areas were 5% less likely to survive cancer compared to their urban counterparts [26]. However, to date there are no studies reporting differences in survival in patients with PM in the US, and few studies have evaluated treatment differences between groups. In our study, patients residing in nonmetropolitan areas were less likely to receive chemotherapy and surgical management, which were associated with decreased hazard of death. While we cannot definitively say whether this is due to patient choices or systemic barriers, the results of previously published studies point toward a systemic issue. One study utilized the National Cancer Database to evaluate differences in surgical management for patients with PM, finding that patients residing in rural areas and those farther from the treating center were more likely to have extrapleural pneumonectomy than lung‐sparing extended pleurectomy/decortication [27]. A number of mechanisms contribute to this disparity, including limited access to healthcare systems and screening tools in these communities [26]. This deprivation of care may also be compounded by the interaction between lower socioeconomic status and rurality [28]. Future studies should assess differences in receipt of immunotherapy between rural and urban patients with PM, as well as the impact of nurse navigators and palliative care referrals on receiving appropriate treatment and combating this disparity.

Our study is similar to that of Bangolo and colleagues, who used the SEER database to assess survival predictors in patients with PM diagnosed between 2010 and 2017 [29]. We found comparable results, with worse survival seen in men, older adults, those diagnosed at advanced stage, and those with fibrous or biphasic subtypes. Our study grouped patients in metropolitan and nonmetropolitan areas, while Bangolo and colleagues utilized multiple geographic identifiers contained in the SEER database. Our results showed a significant 18% higher hazard of death in patients residing in nonmetropolitan areas compared to a 15% higher hazard seen in Bangolo's study that was trending toward significance. Our result may have been significant due to the larger sample size in our study attained by creating larger groups, as evidenced by the fact that the result in Bangolo et al.'s study was trending toward significance, given that most other findings were similar.

We assessed current PM incidence trends in US metropolitan and nonmetropolitan areas, finding that metropolitan areas had higher incidence across the study period. Given that we found a lower average age‐adjusted incidence in nonmetropolitan as compared to metropolitan areas and that previous studies have shown higher concentrations of ambient asbestos in urban versus rural areas, this finding demonstrates a geographic disparity in incidence [8]. Additionally, we found that although incidence rates declined for both groups over the study period, cases in nonmetropolitan areas are declining at a slower rate. It is possible that in addition to reduced access to treatment, individuals residing in nonmetropolitan areas are more likely to have occupations that continue to expose them to asbestos, including industrial and shipyard work [3]. Additionally, it is possible that industrial practices have been slower to enact changes regarding asbestos exposure in these nonmetropolitan areas. We are hopeful that the EPA's 2024 prohibition of chrysotile asbestos and additional research into the impact of social determinants of health on the survival of patients with PM will aid in closing this gap.

This study has a number of strengths and limitations. One strength is that we utilized a validated, population‐based dataset, which ensured accuracy in the study results. This dataset allowed us to generate a large, comprehensive sample size for the study, which is generalizable to the US population. Additionally, we were able to capture longitudinal data regarding incidence and survival in the US using SEER data. Since this is a retrospective review of a publicly available deidentified dataset, granular patient‐level data (including lab values or pathology results) were not available, limiting the study. Additionally, the SEER database does not capture whether each patient received immunotherapy, which has become a recommended first‐line treatment modality for patients with PM, leading to decreased generalizability of the study findings. The SEER database includes fibrous mesothelioma as one histological subtype, which is not used in PM clinical practice but is synonymous with sarcomatoid mesothelioma.

## 5 Conclusion

Our study demonstrates a decrease in PM incidence in both metropolitan and nonmetropolitan areas and improvement in 1‐ and 5‐year CSS between 2004 and 2021 in both metropolitan and nonmetropolitan areas, demonstrating the promising potential impact of the evolving treatment landscape and restrictions in asbestos exposure. However, despite the survival gap narrowing, there is still a difference in survival between metropolitan and nonmetropolitan areas, indicating that more must be done to mitigate these disparities. Clinicians and policymakers alike may continue to improve upon these promising findings and decrease the survival gap by improving patient education, awareness of patient advocacy groups, and increasing access to healthcare services including primary care, palliative care, referrals to centers of excellence, and telehealth resources. Finally, it is vital that all patients with mesothelioma have access to reliable information to make informed decisions about their care and the potential exposures that led to their disease.

## Author Contributions

Conceptualization: A.J.D., L.R. Data curation: A.J.D. Formal analysis: A.J.D. Methodology: A.J.D., L.R. Project administration: L.R. Software: A.J.D. Supervision: L.R. Visualization: A.J.D. Writing – original draft: A.J.D., C.L. Writing – review and editing: M.L., J.G., A.A., J.K., R.M., K.H., P.S., C.R., D.P.C., C.P., D.O., L.R.

## Funding

The authors have nothing to report.

## Conflicts of Interest

The authors declare no conflicts of interest.

## Data Availability

The data that support the findings of this study are available from the corresponding author upon reasonable request.
